# On the Relative Value of Certain Physical Signs

**Published:** 1906-11-24

**Authors:** J. Porter Parkinson

**Affiliations:** Physician to the London Temperance Hospital and to the North-Eastern Hospital for Children


### ON THE RELATIVE VALUE OF CERTAIN PHYSICAL SIGNS.

By J. PORTER PARKINSON, M.D., M.R.C.P., Physician to the London Temperance Hospital and to the North-Eastern Hospital for Children.

The accurate diagnosis of medical diseases depends upon a due consideration of many factors; the history, when obtainable, may be of great value, the symptoms complained of by the patient may also be very important; but physical signs, when present, give usually the most accurate information. It is necessary to combine all the leading facts obtained from these three sources into a consistent whole, which gives then the clue to the nature of the disease from which the patient is suffering. I propose to point out as briefly as possible a few instances illustrating the relative value of some physical signs in a few common diseases such as are met with in every-day practice, and the importance of disregarding (for the purposes of diagnosis) others which frequently tend to confuse the diagnostician.

There are few common diseases which so frequently escape diagnosis as pleural effusion; and this mistake is, I think, due to overlooking the great relative importance of extreme dullness and absence of tactile vocal fremitus. There is an extreme dullness and sense of resistance produced by pleural effusion which is only to be met with to that degree in extensive fibrosis of the lung, in which condition the retraction of the chest prevents as a rule any likelihood of error. The dullness of pneumonia is generally a relative dullness; that of effusion an absolute, wooden dullness. Vocal fremitus is, of course, not always to be obtained over healthy lung in women and children; but when it is present elsewhere, its absence over the dull area is almost pathognomic of pleural effusion. The sign of next importance is displacement of the heart; but this sign is not uncommonly absent even in moderate effusions. For example, last week I removed eleven ounces of fluid from the chest of a child 4½ years old, whose apex beat was in the normal situation. It cannot be too much insisted upon that the breath-sounds over an effusion may be well heard and bronchial in character; this was the case in the child above mentioned, and led the house physician to think the physical signs indicated consolidation of the lung. This character of the breath-sounds is certainly more common in children than in adults, but it is not infrequent in the latter, though absence or weakness of the breath-sounds is more usual.

The voice-sounds are usually weak over an effusion, but sometimes they are well heard, and there may be ægophony below the upper limit of the effusion; but it must be remembered that ægophony is sometimes heard over consolidated lung when no effusion is present.

An effusion at the base of the right side of the chest may be simulated by enlargements of the liver producing an extension of the hepatic dullness upwards; in these cases the following sign will be found to be a valuable distinction: A spot is chosen just below the upper limit of the dullness, and percussion is practised here during a deep expiration and a deep inspiration; if the dullness be due to conditions arising below the diaphragm, the note will vary considerably in pitch and will often become quite resonant if the breath be held at the end of a full inspiration. The same sign may be used in distinguishing between a small effusion in the left axillary region from enlargement upwards of the splenic dullness.

When fluid in the pleura coexists with lung disease beneath, as is so frequently the case when pneumonia causes pleural effusion, the physical signs are, of course, those of the effusion, and the diagnosis of pneumonia depends upon the history and the symptoms of the case, such as the characteristic sputum, the rapid breathing, the type of fever, etc.

The exploring syringe is now so frequently used that students and practitioners are apt to undervalue the preliminary steps to diagnosis; this is to be deplored for many reasons. The exploration of the chest is a proceeding not absolutely without danger; cases occur from time to time where sudden death has followed. Nine such cases have been reported within the last few years, and it is a fair inference that others may have occurred which have not been published.

It is then only right that a careful examination should be made beforehand, and a reasonable suspicion of the presence of fluid be entertained before exploratory puncture is practised. Another reason may also be given; puncture is not a popular proceeding with the lay public, and a patient who is told there is probably fluid in his chest may be unreasonably annoyed to find that the doctor's conclusions are not confirmed.

The physical signs of broncho-pneumonia in children are exceedingly variable, due to the extent and position of the pneumonic areas and to the accompanying collapse. For practical purposes they may be divided into three groups. First, those cases where the patches of consolidation have fused and may extend over the whole or the greater part of a lobe. This is frequent in the primary broncho-pneumonia of young children, but may also occur in broncho-pneumonia secondary to measles, whooping-cough, diphtheria, bronchitis, and the like.

The physical signs in these may be indistinguishable from those of ordinary lobar pneumonia.

Next are those cases in which there is general bronchitis, and, in addition, scattered areas of broncho-pneumonia. Many of these may be too deep in the lung to give any distinctive physical signs, but those which are sufficiently near the surface give rise to bronchial breathing, bronchophony, and crepitations of a peculiar sharp character, very different as a rule from the larger rales due to bronchitis which may be heard elsewhere. The percussion note varies; it may be dullish, but sometimes is hyper-resonant owing to surrounding overdistended lung (acute emphysema), and very frequently a cracked-pot sound is present. It is evident, therefore, that percussion is here of little value, and that preference must be given to the signs heard through the stethoscope.

Finally, there are many cases where there are no consolidated areas sufficiently near the surface to produce characteristic signs, and bronchitis-sounds alone are heard over the chest. Here we have to rely entirely on the symptoms, such as the rapid breathing, the high fever, and the general prostration.

It will be seen, then, that there are three very different types of broncho-pneumonia attended with marked difference in the kind and extent of the physical signs, and that the diagnosis may depend more on certain symptoms than on evidence from examination of the chest, and that in the third variety I have mentioned the physical signs are entirely subordinated to the symptoms. Though slightly irrelevant to the subject of this paper, it may be mentioned that in the not infrequent cases where broncho-pneumonia comes on during the course of whooping-cough, the whoop often ceases.

*Fibrosis of the lung.*—This is by no means an uncommon sequel to pneumonia in children, and the most important signs of its presence are those seen on inspection of the chest. The chest-wall is retracted over its site, and the movements impaired, often to a great extent. The impulse of the heart is displaced towards this retracted side, often to such an extent that in disease of the left lung it may have to be searched for in the axilla or back; or in fibrosis of the right lung it may be displaced to the right of the sternum.

Measurement shows a great diminution in the affected side; vocal fremitus is weak or absent. The percussion note is dull, and may be as flat and toneless as over a pleural effusion; but at places, especially near the angle of the scapula, a tympanitic note may be heard if a dilated bronchus be near the surface; this may be very confusing to the unskilled examiner.

Owing to the drawing upwards of the diaphragm, the percussion note over the lower part of the chest in front and in the axilla on the left side may be tympanitic from the displacement upwards of the stomach; this has sometimes led to the diagnosis of pneumo-thorax; but the displacement of the heart towards the affected side will at once distinguish between the two.

Sometimes this displacement of organs is not present, in spite of the lung being much shrunken. For example, I have a boy of 12 at present under my care who has very considerable retraction of the whole of the right side of the chest, which is almost completely motionless; and yet the heart's impulse is in the normal situation. This is probably due to adhesion of the two pleural surfaces at an early stage of the disease, so preventing the shrinking of the lung to the outer and back part of the chest.

I have tried to illustrate in the above few instances the importance of appreciating the relative value of physical signs in the diagnosis of diseases of the lungs, and similar instances could be given in diseases of the other systems; for example, the immense value of inspection in diseases of the heart or of the abdominal viscera. I feel sure that many mistakes in diagnosis may be avoided by attention to such points; but again I wish to emphasise that no accurate diagnosis can be made without attention being paid to many other details, such as the important points of the history and symptoms. It is useless to multiply instances, as sufficient have been given to illustrate the object of this paper.

